# Neural Dynamics during Binocular Rivalry: Indications from Human Lateral Geniculate Nucleus

**DOI:** 10.1523/ENEURO.0470-22.2022

**Published:** 2023-01-11

**Authors:** Irem Yildirim, Keith A. Schneider

**Affiliations:** 1 Department of Psychological and Brain Sciences, University of Delaware, Newark, Delaware 19716; 2 Center for Biomedical and Brain Imaging, University of Delaware, Newark, Delaware 19716

**Keywords:** binocular rivalry, fMRI, lateral geniculate nucleus, magnocellular, parvocellular

## Abstract

When two sufficiently different stimuli are presented to each eye, perception alternates between them. This binocular rivalry is conceived as a competition for representation in the single stream of visual consciousness. The magnocellular (M) and parvocellular (P) pathways, originating in the retina, encode disparate information, but their potentially different contributions to binocular rivalry have not been determined. Here, we used functional magnetic resonance imaging to measure the human lateral geniculate nucleus (LGN), where the M and P neurons are segregated into layers receiving input from a single eye. We had three participants (one male, two females) and used achromatic stimuli to avoid contributions from color opponent neurons that may have confounded previous studies. We observed activity in the eye-specific regions of LGN correlated with perception, with similar magnitudes during rivalry or physical stimuli alternations, also similar in the M and P regions. These results suggest that LGN activity reflects our perceptions during binocular rivalry and is not simply an artifact of color opponency. Further, perception appears to be a global phenomenon in the LGN, not just limited to a single information channel.

## Significance Statement

Multiple channels of visual information emerge from the retina, but their role in our visual perception remains unclear. Binocular rivalry is an interesting phenomenon in that the separate stimuli presented to each eye remain stable, yet our conscious perception alternates between them. We tested whether both the magnocellular and parvocellular visual streams contribute to binocular rivalry. We measured their activations during binocular rivalry in the human lateral geniculate nucleus, where these two streams are physically disjoint. We found that unperceived information in both streams was suppressed during binocular rivalry, suggesting that both the magnocellular and parvocellular streams have a role in our conscious perception.

## Introduction

**Figure 1.**
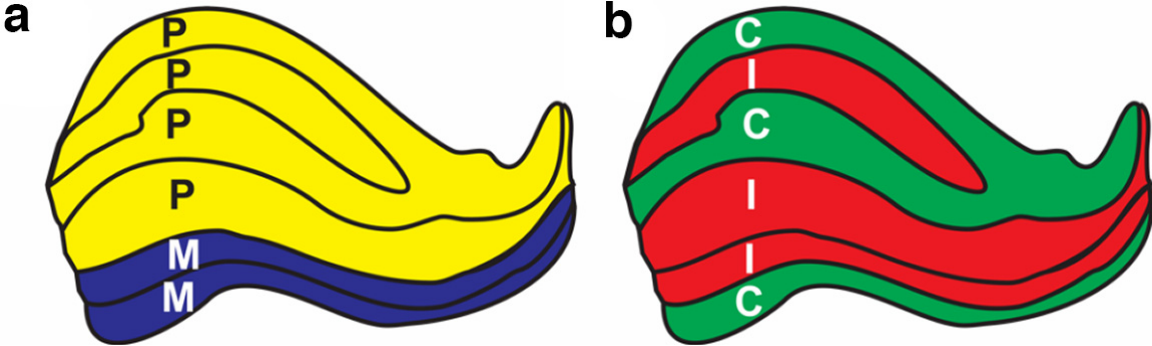
The structure of lateral geniculate nucleus. ***a***, the magnocellular-parvocellular structure; ***b***, the eye-specific structure. C, Contralateral eye; I, ipsilateral eye; M, magnocellular; P, parvocellular. Tracings were generated based on the study by Andrews et al. (1997).

Binocular rivalry (BR) occurs when each eye is presented with a sufficiently different stimulus, resulting in a competition of these monocular stimuli for perception (Alais and Blake, 2015; Blake, 2022), and, typically, alternating perception between the two stimuli. BR provides a means to study visual awareness in the brain (Rees, 2009; Blake et al., 2014), because perception can be decorrelated from the physical stimulus. Here, we investigated the neural mechanisms of BR, focusing on the lateral geniculate nucleus (LGN), the visual relay in the thalamus, and its eye-specific and magnocellular (M) and parvocellular (P) layers (Fig. 1).

In BR, it has been unclear whether the stimuli are competing for perception (Leopold and Logothetis, 1999) or the eyes are competing (Lee and Blake, 1999). Correspondingly, it has been debated whether the visual competition is resolved at the monocular level or at the higher levels in the visual processing hierarchy (Brascamp et al., 2018; Blake, 2022). This debate has settled into hybrid models (for review, see Tong et al., 2006), where the recruitment of different brain regions in rivalry depends on the type of stimuli and the specifics of their presentation.

He et al. (2005) noted that the eye versus stimulus rivalry debate ignored the fact that the visual system operates in parallel streams, including the M and P pathways. They proposed BR as a P pathway phenomenon, based on the psychophysical observations that there is more complete suppression of P pathway stimuli than M pathway stimuli, as indicated by the fewer number of perceptual alternations and/or less piecemeal rivalry or fusion (Levelt, 1965, 1967; O’Shea and Blake, 1986; Burke et al., 1999; Carlson and He, 2000). More direct psychophysical investigation of the two pathways was performed by Denison and Silver (2012). Using an interocular switch (IOS) rivalry paradigm, in which the rivalry stimuli were swapped between eyes in every one-third second (Logothetis et al., 1996), they found that participants more frequently experienced slow irregular perceptual alternations (i.e., stimulus rivalry) than fast regular alternations (i.e., eye rivalry) using their P-tuned stimuli compared with M-tuned stimuli. This suggested that the P pathway was more closely tied to perceptual resolution than the M pathway (He et al., 2005). However, it is difficult to isolate the M and P pathways using different stimuli, and this conclusion has not yet been tested directly by separately measuring the neural activity in the M and P pathways during rivalry.

Two previous studies measured the human LGN activity during binocular rivalry, both demonstrating the modulation of the LGN with perception (Haynes et al., 2005; Wunderlich et al., 2005). We sought to replicate these studies and also eliminate a confound common to both. These studies used colored stimuli and anaglyph glasses to stimulate each eye independently, with no counterbalancing between eyes. For example, the rivaling stimuli used in the study by Wunderlich et al. (2005) were red/green, which can be coded by single color-opponent neurons in the LGN. We wanted to eliminate the possibility that the magnitude of rivalry measured in the LGN could be confounded with color opponent mechanisms. We therefore used achromatic grating stimuli in our study.

Our goal in this research was to directly measure the contributions of the M and P pathways in conventional BR and in IOS rivalry by measuring activity in the disjoint M and P subdivisions of the LGN (Fig. 1*a*). Using functional magnetic resonance imaging (fMRI), we conducted both the conventional BR and the IOS paradigms using the same participants and stimuli, two achromatic gratings rotating in opposite directions. We also compared these activations to physically alternating stimuli with the same temporal sequence (i.e., replay).

## Materials and Methods

### Participants

The three participants for this study (one male, two females; age range, 28–33 years) were the same as in the study by Yildirim et al. (2022). They were compensated at a $20/h rate for their participation. All participants reported normal or corrected-to-normal vision. The participants used the generic sighting tests to determine their dominant eye. The participants S1 and S2 reported right eye (RE) dominance in sighting, while the participant S3 reported left eye (LE) dominance. The protocols for this study and for that by Yildirim et al. (2022) were approved by the University of Delaware Institutional Review Board.

### Neuroimaging

Three 90 min sessions were conducted on 3 separate days. Participants were positioned in a 3 T Siemens Magnetom Prisma MRI scanner with a 64-channel head coil. At the beginning of each session, we acquired a 3D MPRAGE sequence [0.7 mm isotropic voxels; repetition time (TR), 2080 ms; echo time (TE), 4.64 ms; inversion time, 1050 ms; flip angle (α), 9°; field of view (FOV), 210 mm; phase-encoding acceleration factor, 2; scan time, ∼6 min]. All subsequent scans were aligned to this T1-weighted image of each subject and analyzed in their native space (i.e., the T1-weighted image from their first session).

Whole-brain fMRI data were acquired with a multiband EPI sequence with 84 interleaved transversal slices (1.5 mm isotropic voxels; TR, 1500 ms; TE, 39 ms; α, 75°; FOV, 192 mm; bandwidth, 1562 Hz/pixel; phase encoding, Anterior → Posterior; slice acceleration factor, 6), which took 5 min. In each session, there were ∼10 5 min fMRI runs.

### Apparatus

Stimuli for binocular rivalry experiments were presented with a ProPixx (VPixx) projector with a refresh rate of 120 Hz and 1920 × 1080 resolution. Participants wore circularly polarized paper eyeglasses, and a synchronized circularly polarized filter was positioned in front of the projector lens, allowing for the presentation of different stimuli to each eye at 60 Hz. We measured maximum 5% leakage between the left and right filters of the polarized glasses. The experiments were controlled by a Linux computer and prepared in MATLAB, using the DataPixx toolbox for the ProPixx projector, and Psychophysics Toolbox (Brainard, 1997; Pelli, 1997; Kleiner et al., 2007) routines. Participants held a response box (fORPS, Cambridge Research Systems) in their right hand, with their index and middle fingers placed on separate buttons.

### Experimental procedures

During the 5 min fMRI runs, the participants were instructed to fixate on the central dot in the display, around which were presented rotating gratings. The participants were experienced with the experimental procedures, including keeping their eyes on the fixation dot, since S1 and S3 participated in our pilot study and S2 had practiced inside MRI before their participation in this study. Binocular rivalry usually manifests piecemeal over the large stimuli that we needed to use to simulate a significant volume of the LGN and to minimize the effects of fixation errors, but rotation has been shown to aid in the coherent perception of the full-field stimulus (Haynes et al., 2005; Sylvester et al., 2007). Participants continuously reported the perceived rotation throughout each run using a response box by pressing and holding the button under their index finger to indicate counterclockwise rotation, the button under their middle finger to indicate clockwise rotation, or both buttons simultaneously for a mixture perception. There were also six 5 s blank periods presented throughout a 5 min run, during which the participants did not press any buttons. These blank periods consisted of a blank neutral gray screen and were pseudorandomly placed outside the first or last 30 s of the block and separated by at least 25 s of visual stimuli.

**Figure 2.**
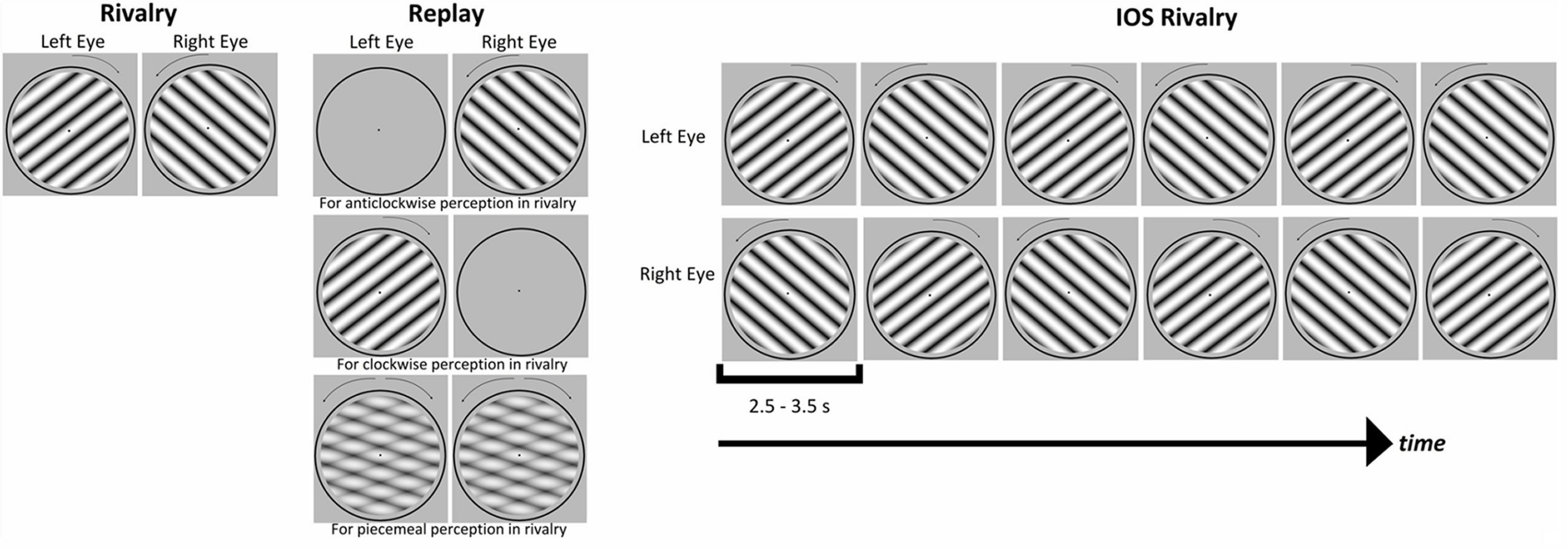
The stimulus presentation for the experimental conditions. Rivalry was the conventional binocular rivalry paradigm in which each eye was presented with a different stimulus, and rotations were counterbalanced across the eyes in the actual experiment. Replay was the physical alternations of the stimuli matching with the perceptual reports of the observer during a previous rivalry block. IOS Rivalry was the interocular switch paradigm in which the rivalry stimuli swapped between the eyes in every few seconds, randomly chosen between 2.5 and 3.5 s.

A 5 min run could be one of the following three conditions: conventional rivalry, replay, or IOS rivalry (Fig. 2). At the beginning of the first session, while the T1-weighted image of their brain was obtained, participants completed a practice conventional rivalry run. The order of the three conditions within a session was fit to a Latin square design, and the starting condition was counterbalanced between participants. However, some runs needed to be repeated because of errors in video frame synchronizations that disrupted the dichoptic presentation and were replaced at the end of the session or at the beginning of the participant’s next session. Across all the experimental sessions, there were 10 runs for each condition for each participant, with the exception that there were 9 rivalry and 9 replay runs for S2 and 8 IOS rivalry blocks for S3.

#### Rivalry

The conventional rivalry condition, hereafter simply referred to as “rivalry,” is illustrated on the left side of Figure 2. Each eye was presented with a grating rotating in opposite directions at ω = 1 cycles/s. Each grating subtended 12° of visual angle in diameter and had a full-contrast sinusoidal profile with a spatial frequency of 7 cycles/°. Each grating was framed by a black circle, measuring 12.5° in diameter to act as a binocular anchor, and was presented on a neutral gray background. The luminance of the blank gray background with the polarization shutter active on the projector was 260.6 cd/m^2^ (model PR655, PhotoResearch) and 100.9 cd/m^2^ measured through either side of the polarization filters that the subjects wore. The rotating stimulus had a projected luminance of 262.7 cd/m^2^; that is, the same as the neutral background. Measured through one of the glasses, it was 70.46 cd/m^2^. The clockwise and counterclockwise gratings were counterbalanced between the eyes across rivalry runs, with the initial directions also counterbalanced across participants.

#### Replay

Replay was a control condition for the conventional rivalry. The participant’s reports from a previous rivalry run were used to mimic their perception. For the first replay run, the perceptual reports from the practice rivalry run were used. This was because of the Latin square design for the order of the conditions where the first replay run was not always preceded by a conventional rivalry run. As illustrated in Figure 2 for replay, to mimic the participant’s clockwise perception in rivalry, the clockwise rotating grating was presented to the corresponding eye while the other eye was presented with the blank instead of a grating. Similarly, if the participant reported counterclockwise rotation in rivalry, then the counterclockwise rotating grating was shown to the matching eye while the other eye was presented with the blank in replay. For the reports of mixture perception in rivalry, the two rotations, superimposed on each other at 50% opacity, were shown to both eyes in replay. The blank screen periods in replay were also matched to the rivalry run.

#### Interocular switch rivalry

The IOS rivalry runs followed the same procedure as the rivalry runs, with the addition of interocular switches of the stimuli (Fig. 2). In the literature, IOS rivalry paradigms used stable orthogonal gratings that flickered at a high frequency (e.g., 18 Hz) and swapped between eyes three times per second (Logothetis et al., 1996; Denison and Silver, 2012). For the current study, we found that flickering the rotating stimuli drastically impaired the perception of rotation; therefore, the stimuli in our IOS rivalry experiments did not flicker but their rotation did present a temporal modulation. The eye swap intervals of the stimuli were determined with the following constraints. First, the swap interval had to be >1 s, the time for a 360° rotation, to allow a stable perception long enough for the participants to respond. Second, the swaps were constrained to occur irregularly to prevent a dull plaid perception of rotations, always touching each other at the same point and rotating backward at regular intervals. Therefore, the gratings in the IOS rivalry runs swapped between eyes at times randomly chosen between 2.5 and 3.5 s.

### Data processing and analyses

We used FSL software (https://fsl.fmrib.ox.ac.uk/fsl/fslwiki/FSL) to process all MRI data unless otherwise noted. Functional data were preprocessed using motion correction via MCFLIRT, intensity normalization, high-pass temporal filtering, and no spatial smoothing.

The fMRI data for each rivalry and replay block were analyzed with a generalized linear model (GLM). Two explanatory variables (EVs), LE perception and RE perception, were defined based on the participant’s perceptual reports. Mixture perceptions were included in both EVs, and the random blank screens were excluded. Motion outlier volumes, identified with the fsl_motion_outliers command thresholded at the 75th percentile plus 1.5 times the interquartile range, were added as a confound. All the possible contrasts were computed between the two EVs. A fixed-effects analysis was used for each participant to combine the multiple scanning runs for each of the rivalry and replay conditions, with all the possible contrasts computed between the conditions. The significance threshold was not corrected for multiple comparisons as the analysis was only performed in the LGN. A suppression index (SI) for each voxel was calculated using the *B* weights provided by the GLM: SI = *B*_rivalry – replay_/*B*_replay + rivalry_ where *B*_rivalry – replay_ is the contrast between the rivalry and replay runs, and *B*_replay + rivalry_ is their conjunction. An index of 0 would indicate complete suppression during rivalry, wherein voxels would exhibit similar amplitudes to those induced by the physical stimulus alternations in replay, whereas a significantly positive index would indicate that voxels in the LGN were still activated by the nonperceived stimuli.

The fMRI data for the IOS rivalry condition were not processed, as stimulus rivalry was not observed in any participant (see Results).

### Regions of interest analyses

The regions of interest (ROIs) in this study were the M and P regions and the eye-specific regions in the LGN. For the M and P comparisons, the LGNs were anatomically identified for each participant separately using quantitative MRI, and the M and P regions in each LGN were segregated based on their difference in T1 relaxation time, which was reported in the study by Yildirim et al. (2022). For the eye-specific region comparisons, the anatomically identified LGNs were adjusted for the visually active areas using a visual hemifield stimulation. The contralateral (CL) and the ipsilateral (IL) eye regions were then segregated based on their responses to the eye-specific visual stimulation in both monocular and dichoptic viewing conditions. Eye-specific voxels were determined as those that exhibited a significant contrast between eyes in the combined GLM analysis of the monocular and dichoptic eye localizers, as detailed in the study by Yildirim et al. (2022).

The GLM results for the rivalry–replay contrast were inspected in the M and P sections of each LGN, and the suppression index was compared between the regions. In addition, an event-related averaged time series was computed in MATLAB. For each of the eye-specific voxels in each LGN (Yildirim et al., 2022, their Fig. S1), the preprocessed fMRI data were converted to the percentage change using the mean baseline activity, corresponded to the blank screens, and corrected 6 s for the hemodynamic delay. The data were then upsampled in time to 100 ms resolution (from the original TR of 1.5 s). For each event, corresponding to a participant’s indication of a single rotation direction, the event-related average was calculated between 5 s before and 15 s after the participant’s initial response. This analysis was carried for the eye-specific regions in the LGN but not separately for those in the M and P regions because the contralateral M layer could not be identified by the eye localizer tasks (Yildirim et al., 2022, their Fig. S1).

## Results

### Perceptual findings

**Figure 3.**
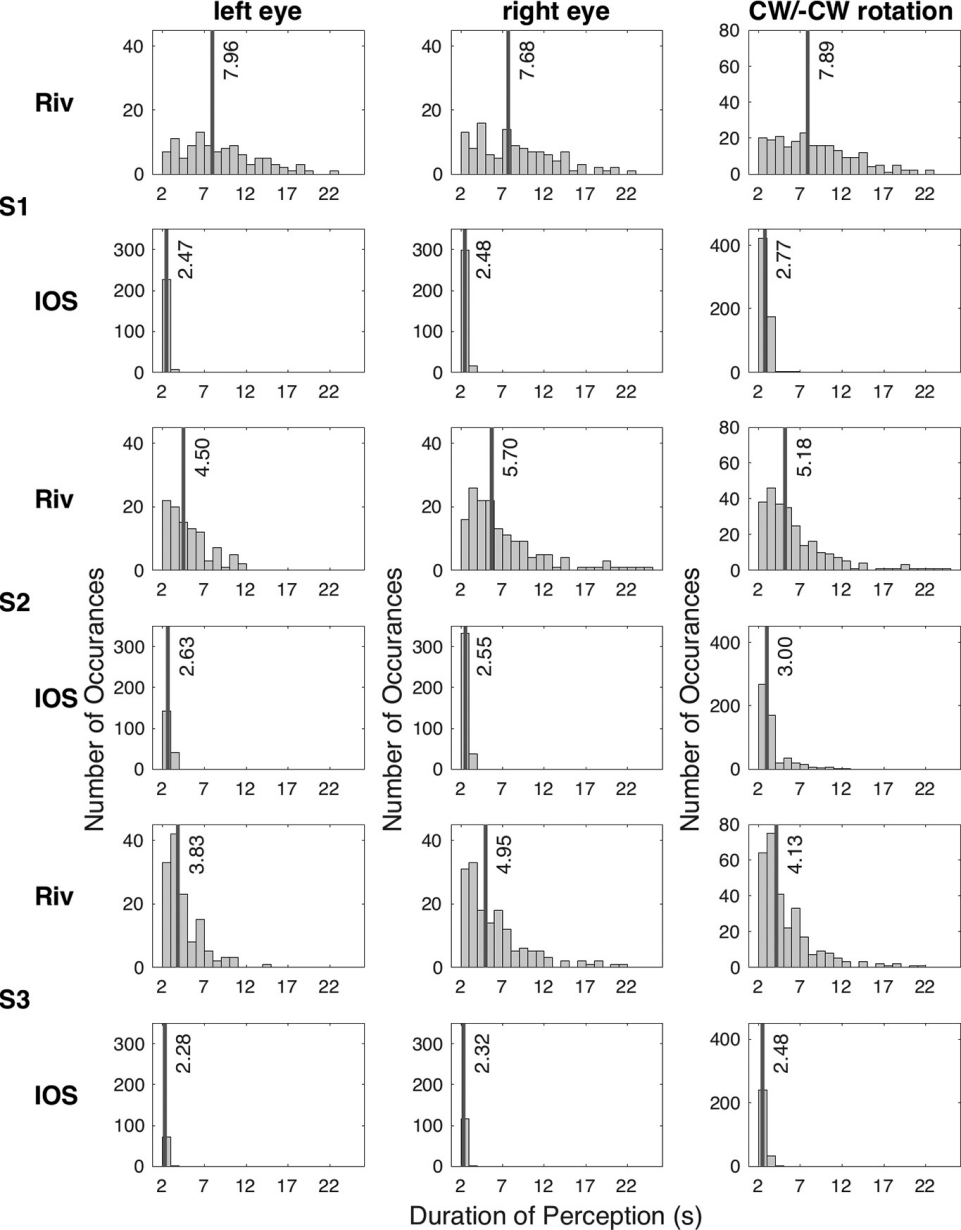
Histograms for durations of exclusive perceptions during rivalry. Riv, Rivalry; CW, clockwise rotation; –CW, counterclockwise rotation. In the third column are the histograms collapsed across the two rotations for exclusive perceptions. Black lines reflect the median duration. Bin interval is 1 s.

We calculated the durations of unmixed perception of a single rotation, corresponding to a single eye, based on the participants’ perceptual reports. These are shown in Figure 3, for perceptions lasted >2 s, concatenated across all scanning runs for the rivalry conditions. As designed, the rivalry and the replay conditions resulted in similar perceptual reports, since the replay stimuli were constructed to mimic the perception of the rivalrous stimuli. The average unmixed perception during rivalry across participants was ∼7 s, with means ranging from 4.45 to 8.81 s, and the average median value was 5.77 s, with medians ranging from 3.83 to 7.96 s (Fig. 3, black lines for rivalry). The durations of the perceived clockwise versus counterclockwise rotations did not differ for any participant during rivalry. However, RE perceptions were longer than LE perceptions for two of the three participants [S2: *t*_(257)_ = 4.12, *p*_Bonferroni_ < 0.001, *d *=* *0.52; S3: *t*_(292)_ = 4.23, *p*_Bonferroni_ < 0.001, *d *=* *0.49]. During IOS rivalry, on the other hand, one of these participants (S2) experienced longer LE perceptions instead (*t*_(551)_ = 3.46, *p*_Bonferroni_ = 0.002, *d *=* *0.31) and the other participant (S3) experienced longer clockwise perceptions (mean* *=* *2.59, SD =* *0.37) compared with the counterclockwise perceptions (mean* *= 2.48, SD* *=* *0.35; *t*_(272)_ = 2.43, *p*_Bonferroni_ = 0.047, *d *=* *0.29).

As can be seen in the third column in Figure 3, the perceptual reports demonstrated no stimulus rivalry in the IOS condition. Neither the number of occurrences of exclusive perceptions (*y*-axis) nor the perceptual durations (the distribution and the black lines) during IOS rivalry were similar to the rivalry condition. In fact, the perception failed to be resolved on a single rotation, or on a single eye, during IOS rivalry. To quantify the amount of eye rivalry, stimulus rivalry, and the mixture of perception, we focused on when the interocular switch (i.e., eye swap) of the stimuli occurred. If the eye swap went unnoticed, indicated by the exclusive perception of a rotation by the time the swap had happened, we counted it as stimulus rivalry. On the other hand, if the eye swap was noticed, indicated by the initiation of an exclusive perception following the eye swap within 500 ms, we counted it as eye rivalry. There was neither eye rivalry nor stimulus rivalry, instead, the reported perception was mostly the mixture of the two rotations for the vast majority of the eye swaps: 99.57% for S1, 99.68% for S2, and 100% for S3. Given our failure to induce IOS rivalry, the data for this experiment were not analyzed further.

### Rivalry in the M and P regions of LGN

**Figure 4.**
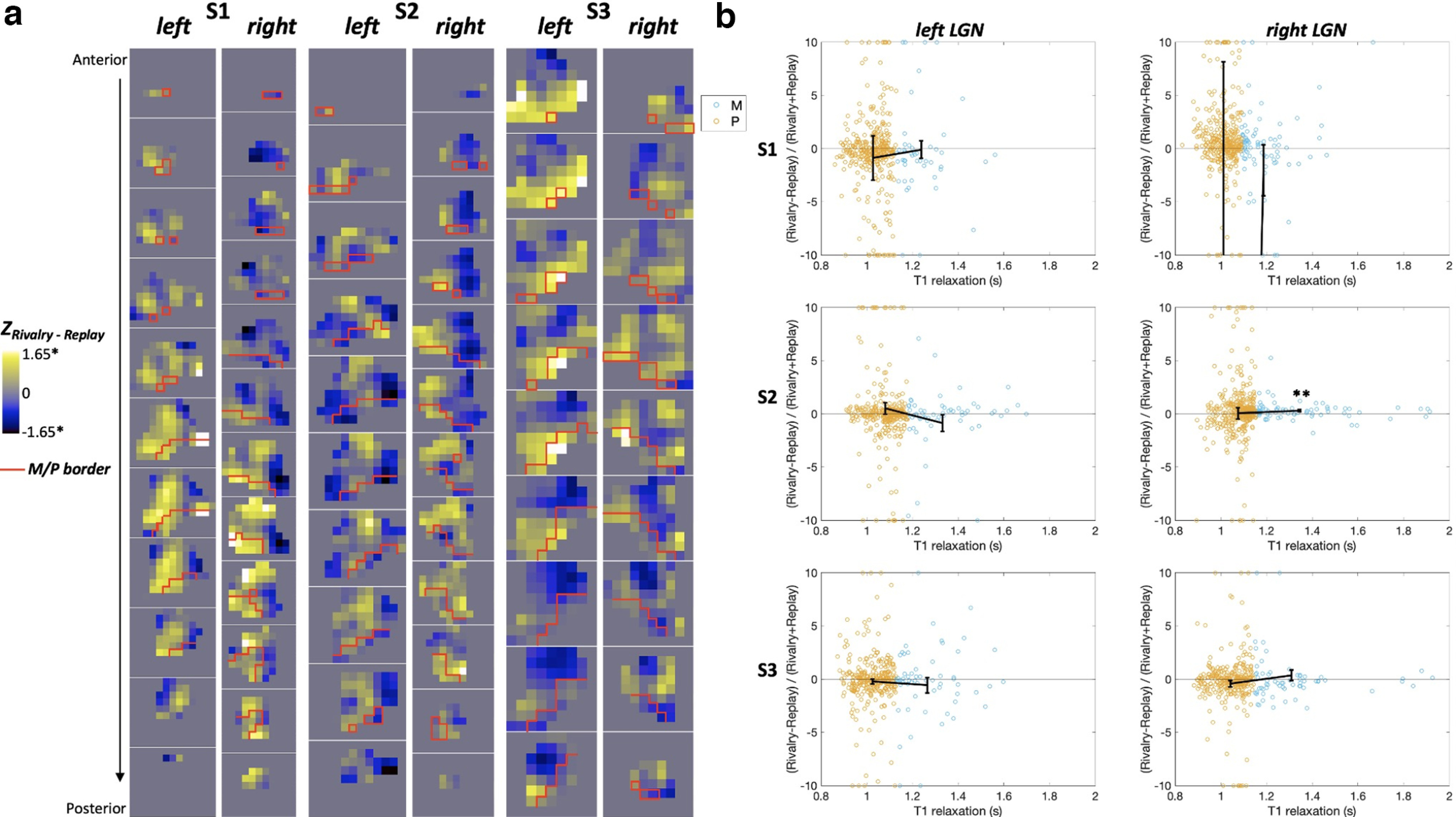
GLM results for rivalry and replay in the M and P regions of LGNs. ***a***, Coronal slices of each LGN showing the *z* scores for the rivalry−replay contrast. The red line delimits the boundary between the M and P regions according to their T1 relaxation time (Yildirim et al., 2022). *p < 0.05, uncorrected for multiple comparisons for voxels. ***b***, Scatterplots of the M and P voxels, indicated by their T1 relaxation time in seconds on the *x*-axis, showing similar suppression for rivalry and replay. The suppression index on the *y*-axis was calculated by the *B* weights. For illustration purposes, the voxels with indices beyond 10 and −10 were plotted at 10 and −10, respectively. Black lines indicate the mean suppression indices for the M and P voxels. Error bars are the SEM. **p = 0.02, Bonferroni corrected for six LGNs.

The contrasts between the rivalry and replay conditions were examined with a GLM within each LGN. As indicated by the *z* scores for the rivalry–replay contrast in Figure 4*a*, rivalry and replay activated similar number of voxels within each LGN and their M and P regions. There were small numbers of voxels that were significantly more active during rivalry (white voxels) or during replay (black voxels). As can be seen in Figure 4*a*, these significant voxels were spread evenly across the M (area below the red line) and the P regions (area above the red line). However, the distribution of rivalry-related and replay-related activity in the LGN looked similar to the retinotopic map in LGN, representing the visual eccentricity. That is, replay (Fig. 4*a*, blue areas) activated regions representing the central field, whereas rivalry (Fig. 4*a*, yellow areas) activated regions representing the surround field (but see Discussion).

In addition, there were similar levels of overall suppression in each LGN, demonstrated by the suppression indices at ∼0 [Fig. 4*b*; i.e., the unperceived stimuli did not increase activity in the LGN, whether it was present (in the rivalry condition) or not (in the replay condition)]. We conducted a Bayesian one-sample *t* test for each LGN and the M and P divisions to see the likelihood of data favoring the null distribution (i.e., index not different from 0, indicating successful suppression) over the alternative distribution (i.e., index different from 0). The odds were in favor of the null hypothesis, at least moderately, across all LGNs, Bayes factor (BF01) values > 8 for LGNs, BF01 values > 5.5 for P regions, and BF01 values > 5 for five of six M regions. The only ROI in which the Bayesian factor favored the alternative hypothesis was the M section of the right LGN in S2 (BF10* *= 11.8). The suppression index was positive in this M section, indicating higher relative activation during rivalry and thus incomplete suppression (Fig. 4*b*). Last, a Bayesian independent-samples *t* test was used on the indices to find how likely it is that the data support the alternative hypothesis that the suppression index is different in the M and P sections of the LGN over the null hypothesis of no difference. Bayesian factors revealed that the odds were moderately in favor of the null hypothesis (BF01 values > 3.5 for all LGNs), indicating similar levels of suppression in the M and P sections.

### Rivalry in the eye-specific regions of LGN

**Figure 5.**
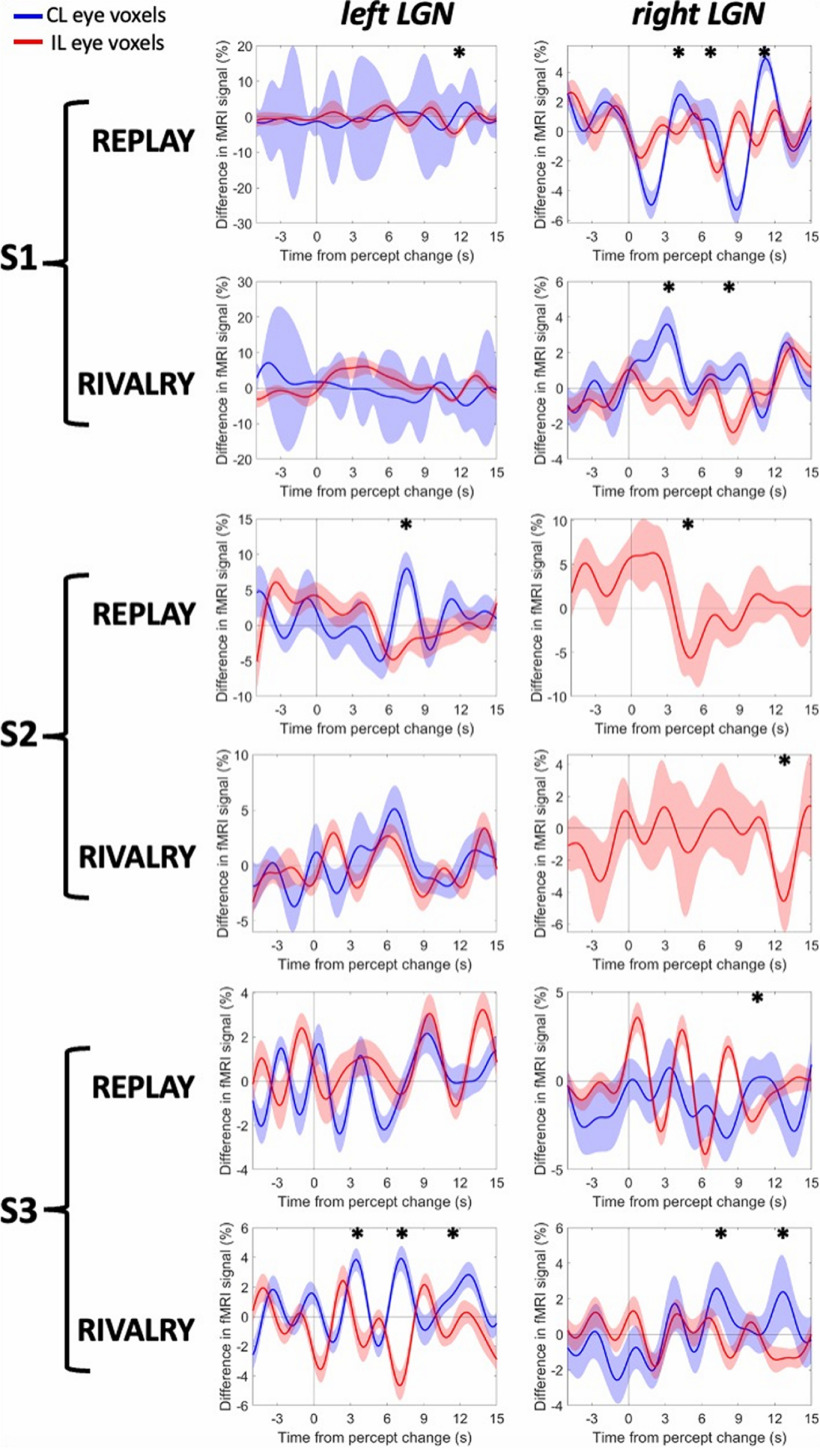
fMRI activity around the exclusive eye perceptions during rivalry and replay. The *x*-axis shows the time around when the stable perception started (time 0), indicated by the participant’s perceptual reports. The *y*-axis shows the difference in the percentage fMRI signal for the CL eye perceptions versus IL eye perceptions. Error shades are 95% CIs. *p < 0.05.

The event-related averages of the CL and IL voxels in each LGN, registered at the beginning of the participants’ report of stable perception (with durations ≥ 6 s; i.e., four TRs of 1.5 s each), are shown in Figure 5. We examined the differences in signal for the CL eye voxels (blue line) and the IL eye voxels (red line), indicating that the CL voxels (IL voxels, respectively) showed increased activity following the perceptual resolution on the CL (IL) eye while showing suppressed activity following the perceptual resolution on the IL (CL) eye.

As can be seen in Figure 5, rivalry and replay conditions did not yield similar results. The rivalry condition resulted in more separable oscillations of eye-specific voxels for three LGNs (S1, right LGN; S3, left and right LGNs), all of which were also more reliable in their eye-specific region analysis, as reported in the study by Yildirim et al. (2022). Focusing only on these LGNs in Figure 5, the oscillations demonstrated signs of the exclusive perception earlier and longer for the rivalry condition than for the replay condition. The LGNs that had few numbers of significant eye-specific voxels (S1, left LGN; S2, right LGN; Yildirim et al., 2022) showed more variance, as evident by the larger 95% confidence intervals (CIs) in the figure.

## Discussion

We measured the relative activities of the M and P pathways in the human LGN during binocular rivalry to test the predictions that they might differ (He et al., 2005; Denison and Silver, 2012). Using fMRI, we observed similar overall responses in the disjoint M and P layers to perceptual alternations induced by rivalry or replay, failing to support this hypothesis. By using polarizing filters to present achromatic stimuli dichoptically, we also confirmed that binocular rivalry can be observed in the human LGN and is not simply an artifact of color opponent processes. As discussed in the study by Yildirim et al. (2022), the contralateral M layer did not appear reliably; therefore, the comparison of M and P was made in overall activity but not in the time series activity. Our attempts to induce rivalry in the IOS paradigm was unsuccessful using our stimuli, and we were therefore unable to test whether stimulus rivalry was associated with the P but not the M pathway (Denison and Silver, 2012).

We used stimuli that did not specifically bias M or P neurons so that any differential activity between them is not because of stimulus and found similar activations during rivalry and replay for the M and P layers of LGN. However, we did observe a distribution of rivalry-related and replay-related activity in the LGN (Fig. 4*a*) that resembled the retinotopic map in LGN representing the visual eccentricity (Schneider et al., 2004; DeSimone et al., 2015), such that replay activated areas that corresponded to central field and rivalry activated areas that corresponded to the surround field of the stimulus. One possible explanation could be that eye movements with participants being cross-eyed during rivalry could cause changes in peripheral activation. However, this is unlikely, as our experienced participants were trained to fixate and our stimuli were full field, which by design are less susceptible to small changes in fixation. Another explanation for the potential retinotopic pattern could be the difference in the piecemeal perception induced by rivalry and replay. The large rivalry stimuli might have resulted in different rotations at center and surround, reported as a mixture perception by the participants, whereas the corresponding replay stimulus was homogeneous and weaker because the rotations were superimposed at half opacity. However, we did not localize the retinotopic maps with an eccentricity manipulation in the visual stimulus; thus, any conclusion requires additional research.

Using rotation-opponent achromatic gratings, we observed eye-specific responses to the exclusive eye perceptions that were not as pronounced or prolonged compared with those in the study by Haynes et al. (2005). They used orthogonal blue/red gratings rotating in the same direction. It is possible that rivalry between constantly orthogonal gratings supported longer perceptions (Blake et al., 2003), whereas the motion opponency in our study prevented building coherent perception. In our stimuli, the gratings were only briefly orthogonal as they rotated in opposite directions, and thus their rivalry was not based on spatial orthogonality. The mean duration of the exclusive perception in our study was ∼2 s shorter. However, Haynes et al. (2005) did not give an option to report mixture perception, so it is difficult to compare with our study precisely.

We found in some participants that the perceptual reports during rivalry occurred earlier than during replay. This would be consistent with the study by Wunderlich et al. (2005) and might reflect the buildup activity in the monocular neurons preceding perceptual resolution (Wolfe, 1986; Lehky, 1988; Blake, 1989; Tong, 2001). Surprisingly, the eye-specific activity in the replay condition was noisier than during the rivalry condition. We speculate that this could be driven by factors such as reaction time noise or motion adaptation and the resulting motion aftereffects during replay. Also, the monocular neurons in the LGN receive binocular feedback from primary visual cortex (Dougherty et al., 2021), and fMRI measurements may have different confounds in the two conditions through feedback and lateral connections (Dougherty et al., 2019). During rivalry, for example, it might be the feedback information that is being suppressed. Unperceived information could still be transmitted feedforward, but the feedback activity would be required for conscious perception (Pascual-Leone and Walsh, 2001). This could also explain previous findings indicating sensitivity to the changes in the suppressed stimulus even if it might be reduced (Wales and Fox, 1970).

We observed that two of three participants showed an RE dominance during rivalry, as indicated by the longer exclusive perceptions for the RE than the LE. These participants reported having different dominant eyes in the simple sighting tests. Correlations between the dominant eye in sighting and the dominant eye in perception during rivalry have been reported previously (Porac and Coren, 1978; Handa et al., 2004); however, it has been also suggested that BR is sensitive to the sensory eye dominance and not sighting eye dominance (Dieter et al., 2017). Our results, albeit with a small sample, support this latter conclusion.

When the rotating gratings swapped between eyes during the IOS rivalry experiment, mixture perceptions dominated. Given that neither stimulus dominated, this suggests that both stimulus and eye competition occurred. Previous research with the IOS paradigm used orthogonal gratings that rapidly swapped between eyes while also flickering rapidly to mask the eye swap. This results in more stimulus than eye rivalry (Logothetis et al., 1996; Lee and Blake, 1999; Silver and Logothetis, 2007; Bhardwaj and O’Shea, 2012; Denison and Silver, 2012; Patel et al., 2015). Christiansen et al. (2017) found that the IOS rivalry stimuli that differed only in color resulted in stimulus rivalry, whereas stimuli that differed in luminance did not. This is consistent with our finding of no stimulus rivalry using the achromatic stimuli in our study. This also suggests that the M stimuli might not be optimal for generating slow stimulus alternations (He et al., 2005; Denison and Silver, 2012).

Sandberg et al. (2011) used an eye-swapping procedure with flickering stimuli and found that continuous perception was not disrupted by the eye swaps between the complex stimuli (i.e., faces/houses) but was for simple stimuli (i.e., orthogonal gratings, not rotating). This was the case even when they scrambled their complex stimuli, suggesting an importance for overlapping low-level features. Our rotating stimuli that continually changed the overlapping features may therefore not be optimal to resolve the competition with the eye-swapping procedure.

In conclusion, this study demonstrated that binocular rivalry could be observed in the human LGN using achromatic rotation–opponent rivalry stimuli. We observed nearly complete suppression of the nonperceived eye activity in both the M and P regions, and a different time course of activity between rivalry and the actual physical alternation of the different stimuli, despite similar perceptual experiences.

## Synthesis

Reviewing Editor: Ifat Levy, Yale University School of Medicine Decisions are customarily a result of the Reviewing Editor and the peer reviewers coming together and discussing their recommendations until a consensus is reached. When revisions are invited, a fact-based synthesis statement explaining their decision and outlining what is needed to prepare a revision will be listed below. The following reviewer(s) agreed to reveal their identity: Michael Schmid. Note: If this manuscript was transferred from JNeurosci and a decision was made to accept the manuscript without peer review, a brief statement to this effect will instead be what is listed below.

Investigations of binocular rivalry are important to understand the neural basis of visual awareness. Previous research has demonstrated involvement of the early stages of the visual system, including LGN. Further clarification of the LGN pathway contributions is a significant step forward.

The authors have adequately addressed previous comments.
